# Estimating bulk density of compacted grains in storage bins and modifications of Janssen's load equations as affected by bulk density

**DOI:** 10.1002/fsn3.23

**Published:** 2013-01-07

**Authors:** Ekramul Haque

**Affiliations:** Department of Grain Science and Industry, Kansas State UniversityShellenberger Hall, Manhattan, Kansas, 66506

**Keywords:** Compaction, density, grains, Janssen, loads storage

## Abstract

Janssen created a classical theory based on calculus to estimate static vertical and horizontal pressures within beds of bulk corn. Even today, his equations are widely used to calculate static loadings imposed by granular materials stored in bins. Many standards such as American Concrete Institute (ACI) 313, American Society of Agricultural and Biological Engineers EP 433, German DIN 1055, Canadian Farm Building Code (CFBC), European Code (ENV 1991-4), and Australian Code AS 3774 incorporated Janssen's equations as the standards for static load calculations on bins. One of the main drawbacks of Janssen's equations is the assumption that the bulk density of the stored product remains constant throughout the entire bin. While for all practical purposes, this is true for small bins; in modern commercial-size bins, bulk density of grains substantially increases due to compressive and hoop stresses. Over pressure factors are applied to Janssen loadings to satisfy practical situations such as dynamic loads due to bin filling and emptying, but there are limited theoretical methods available that include the effects of increased bulk density on the loadings of grain transmitted to the storage structures. This article develops a mathematical equation relating the specific weight as a function of location and other variables of materials and storage. It was found that the bulk density of stored granular materials increased with the depth according to a mathematical equation relating the two variables, and applying this bulk-density function, Janssen's equations for vertical and horizontal pressures were modified as presented in this article. The validity of this specific weight function was tested by using the principles of mathematics. As expected, calculations of loads based on the modified equations were consistently higher than the Janssen loadings based on noncompacted bulk densities for all grain depths and types accounting for the effects of increased bulk densities with the bed heights.

## Introduction

We owe a debt of gratitude to Janssen and his research contemporaries for laying a firm foundation for the discipline of particulate solids and loadings imposed by bulk solids (Roberts 2000). Transmission of pressures created within a mass of matter is dependent on the fundamental character of matter. Fluids and solids transmit pressures differently; in fluids, the pressure at any point is equal in all directions. Bulk solids (granular materials) are called semifluids. They behave neither like fluids nor like solids and display some properties of solid (transmits pressure downward) and some properties like fluid (transmits pressure sideways also). They are compressible to an extent depending on the characteristics of the bulk material, bins containing them, and some external factors. One particular property that affects the estimation of mass in bulk storage is the accuracy of estimation of average-specific weight (bulk density or test weight) within the storage bins. Estimation of mass based on laboratory measurement data of bulk density and the storage volume results in a gross underestimation.

To reduce the estimation error, United States Department of Agriculture (USDA), for example, developed a set of Packing Factor data for grains in the middle of the last century. These data are still used to settle, for example, insurance claims and the value of the stored crops for various financial and other legal transactions. Even though the estimation is based on some known factors affecting it, the procedure is highly empirical and cumbersome. Above all, the estimation results in high levels of error. USDA Risk Management Agency (RMA) entered into a cooperative agreement with USDA Center for Grain and Animal Health Research, Kansas State University, University of Kentucky, and University of Georgia to develop improved methods of estimating Packing Factor and inventory estimation in bins storing barley, corn, grain sorghum, oats, soybeans, and wheat. This article was a part of this effort.

## Objective

The main objective of this article was to develop a mathematical model for estimating the mass of packed beds of cereal grains in storage bins. The model should lay down the basis for developing a fundamental understanding of the variation in the bulk density of stored granular materials due to packing of grains. As an extension, a secondary objective was set to suggest modification of Janssen's equations for calculating vertical and horizontal stresses on storage bins of packed bed of granular materials as affected by the increases of bulk densities due to compaction.

## Literature Review

The first significant published study on the loadings of free-flowing granular materials was done by Janssen (1895). Since Janssen, many studies were conducted, and results were published in various scientific journals of the world. Many standards such as American Concrete Institute (ACI) 313, American Society of Agricultural and Biological Engineers EP 433 (ASABE 2001), German DIN 1055, Canadian Farm Building Code (CFBC), European Code (ENV 1991-4), and Australian Code AS 3774 have been developed to design grain storage bins and systems. Most of these standards are based on Janssen's equations for calculating loads on floors and walls using different constant values of parameters of the equations such as bulk density, friction coefficient, and ratio of horizontal to vertical loads of the stored materials.

However, it is widely recognized and accepted that material bulk density in grain bins does not remain constant, but increases with the bed depth. Attempts have been made by many including Thompson and Ross (1983), Malm and Backer (1985), Thompson et al. (1987, 1990, 1991) to determine Packing Factors and compressibility of different cereal grains. ACI standard (1997) for design pressures in grain bins utilizes the Janssen equation of static equilibrium, multiplied by an overpressure factor to predict horizontal and vertical sidewall loads. Thompson and Ross (1983) measured changes in bulk density by varying the internal pressure and moisture content and found that for moistures in between 8% and 12% the bulk density differed very little, but the largest change occurred at pressures below 14 kPa and also at low overburden pressures on 16–20% moisture wheat. They developed statistical models based on empirical measurements relating bulk density to vertical pressure and moisture content. Malm and Backer (1985) also developed a statistical model relating compaction factor for six cereal grains including barley, hard red spring wheat, durum wheat, oats, oil sunflower, and confectionary sunflower to test weight, dockage, moisture content, and bin area and depth. Bates (1925) recognized that packing of granular materials was a function of depth of material in a bin and different packing factors would exist for different grains. Thompson et al. (1987) used differential form of Janssen's equations to predict variations of bulk densities of stored materials. Packing factors for six different whole grains were estimated by them as a function of moisture content and test weight of stored grains in bins of any height and diameter. A computer model for predicting packing factors of whole grains in flat storage structures was developed by Thompson et al. (1990). Thompson et al. (1991) found that a change in grain height had a greater effect on the packing factor than the change in diameter or moisture content. Using Janssen's approach, Ross et al. (1979) developed a computer technique to estimate the wall pressures and average bulk densities of materials in bins. They allowed grain bulk density, material friction coefficient, and ratio of lateral to vertical pressures of grain to vary within bin as functions of vertical pressure and moisture content. They used empirical equations for bulk density and ratio of lateral to vertical pressures developed in an earlier study (Loewer et al. 1977).

Haque (2011) developed a mathematical equation between the void fraction of grain mass and the depth of packed beds of granular materials with the void fraction decreasing with grain depth. The void fraction depended on the bulk-density function which increased with the bed depth. He applied this equation to Ergun's (1952) pressure drop equation and developed a mathematical function for pressure drops in grain beds that reflected the affect of decrease of void fraction with the bed depth. He demonstrated that in conformity with practical observations, these pressure drops were higher than Shedd (1953) pressure drop values for cereal grains.

This article deals with a theoretical approach relying on a mathematical law of exponential function (e.g., Janssen equation) that states that the first derivative of any exponential function is also exponential. This mathematical property was used to derive a function for bulk density of grain in bins that was dependent on the vertical pressures of grain in bins which in turn depended on the depth of grain. Grain bulk density was then allowed to vary with the depth of grain to derive proposed modifications of Janssen's equations for vertical and horizontal pressures in grain bins.

## Mathematical Model Development and Validation

The fundamental difference between Janssen's load equations and the equations proposed in this study was the basis that Janssen assumed a constant bulk density, whereas in this study, it was assumed to be a function of the bed height. In fact, Janssen's assumption that the bulk density is constant is flawed and results in underestimation of loads in storage bins. To conform to the practical need, various overpressure factors are applied to Janssen's equations.

**Figure 1:**
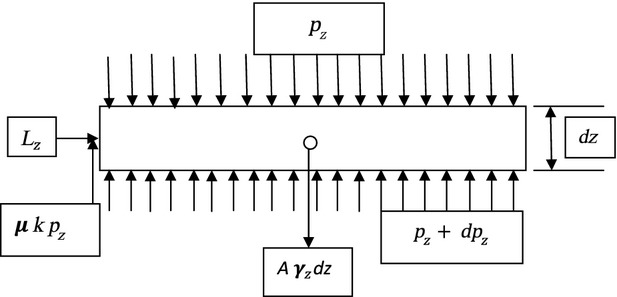
Forces on an elementary volume of granular material in a storage bin.

Like Janssen, let us analyze the forces on an elementary volume of grain mass in a bin (Fig. 1). Let *z* be the vertical direction of the bin, with *z* = 0 being the grain surface and *z* = *z* being any depth of grain in the bin. Also, assume the following symbols: cross-sectional area A; hydraulic radius (cross-sectional area/perimeter) *R*_h_; vertical pressure *p*_*z*_; specific weight of bulk material γ_*z*_ that varies with the depth *z* in grain bin; material friction coefficient μ; and lateral pressure of bulk material *L*_z_. Also, like Janssen let us assume the ratio between lateral and vertical pressure as a constant, *k*.

Summing all forces, “*F*” in the vertical direction *z* equals to zero:


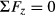
(1)


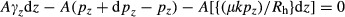
(2)


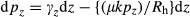
(3)


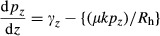
(4)


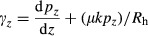
(5)


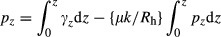
(6)

Now, let us make an assumption based on our existing knowledge of loadings imposed by granular materials and fundamentals of mathematics. Let us assume that the vertical pressure of bulk solids is an exponential function (Janssen 1895) given by the following general equation:


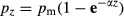
(7)

where 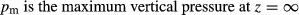 is the maximum vertical pressure at *z* = ∞, α is a constant dependent on properties of bulk solids, the storage bins, and other external factors such as filling methods and vibrations.

As *p*_z_ is exponential as denoted by equation (7), mathematically, d*p*/d*z* must also be exponential. So, the specific weight function, γ_*z*_*,* given by the equation (5) must also be exponential, because equation (5) is the summation of *p*_*z*_ and d*p*/d*z* each of both addends being exponential. We can conclude that γ_*z*_ would also be an exponential function of *z* and we could assume the function to be as shown in equation (8) below:


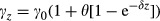
(8)

where γ_0_ is a reference-specific weight, for example, specific weight of the bulk solid at the surface of the bed *z* = 0, that is, laboratory measurement value of the specific weight, and γ_*z*_ is specific weight of the bulk solid at any depth of bed *z* = *z*. θ and δ are constants dependent on properties of bulk solids, the storage bins, and other external factors such as filling methods and vibrations.

It follows from equation (8) that the specific weight is maximum at *z* = ∞ and given by


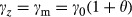
(9)

As both equations (8) and (5) have the same left side, the exponent of e must be equal in both equations (7) and (8), and as such, we could assume α = δ. Equation (8) takes the following form:


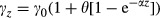
(10)

If the analysis based on our assumption of equation (7) could prove to lead back to the equations (5) and (6), then we could conclude that our assumption was true and valid. To do that, let us prove that the right side of equation (6) will indeed yield the vertical pressure function, *p*_*z*_ the left side of equation (6).

The first term of the right side of equation (6) is


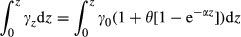
(11)

The solution of equation (11) is


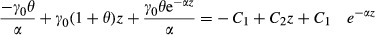
(12)


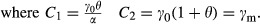


Replacing for *p*_*z*_ from equation (7), the second term of the right side of equation (6) becomes


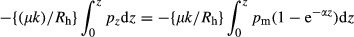
(13)

The solution of equation (13) is


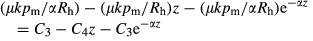
(14)

where *C*_3_ = *μkp*_m_/*α R*_h_ and *C*_4_ = *μ*k*p*_m_/*R*_h_.

Adding both terms of right side of equation (6), that is, adding equations (12) and (14), we get


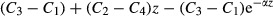
(15)


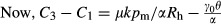


Replacing for *p*_m_ = *γ*_m_ *R*_h_/*μ k*, 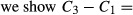 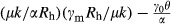



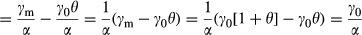
(16)


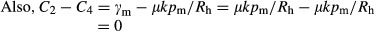
(17)

So, equation (15) reduces to


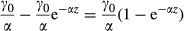


where 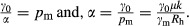



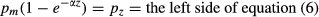
(18)

This clearly proves that the assumption we made was indeed true and valid; the equation (6) was a valid equation representing the vertical pressure imposed by the granular material in a bin, and the specific weight of bulk solids such as cereal grains in bins varied according to the equation (10).

So, it follows obviously that the equation (18) reduces to the following:


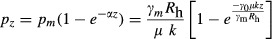
(19)


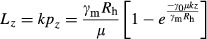
(20)

The equations (19) and (20) are the modified Janssen's equations developed in this study for vertical and horizontal load calculations of free-flowing granular materials stored in deep bins.

If the bulk density is assumed to be constant (γ_m_ = γ_0_ = γ), equations (19) and (20) reduce to the following equations (21) and (22) which are Janssen's equations for vertical and horizontal pressures, respectively.


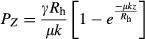
(21)


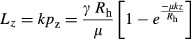
(22)

## Analysis and Results

A mathematical expression describing compaction in a packed bed of granular materials has been developed. It is a common knowledge that the specific weight of bulk solids in a bed has an upper limit which occurs at a large depth of bed, and the lowest value of specific weight is at the surface. The specific weight function developed in this study depends on four fundamental parameters, θ, α, γ_0_, and γ_m_. These parameters are not only interrelated but also carry information about the properties of the bulk solids, geometrical, and other characteristics of the bins such as size, shape, wall roughness, and other external variables, such as filling method and vibration. It is proposed that a set of data for each type of bulk solids of interest is fitted into equation (10) to determine the values of the four fundamental parameters by using different properties of bulk solids, bin characteristics, and some external variables, if necessary. Properties of bulk solids could include laboratory measurement of specific weight with associated amount of foreign and fine materials and moisture content. Bin characteristics could relate to bin dimensions including hydraulic radius and wall materials. After the values of the four fundamental parameters are determined, a set of standards for design and other considerations could be developed. The standards could cover ACI, American Society of Agricultural and Biological Engineers (ASABE), and other international standards.

To study the effects of the proposed modifications of Janssen's equations on the calculated values of loads, two different grains, wheat and oats, were assumed, because wheat is among the heaviest and oats is among the lightest of grains, and also the known compaction characteristics of both grains are on opposite sides of the range.

Let us consider the load calculations on a 9.144 m diameter, 38.1 m high (30 × 125 feet) concrete bin, a size which is quite common. Assuming μ and *k* values 0.40 and 0.50, respectively (ASABE EP 433 Standard for wheat), both Tables 1 and 2 are calculated. For wheat and oats, respectively, an initial bulk density, γ_0_ of 801 and 512.7 kg per cubic meter (50 and 32 lbs per cubic foot), and maximum bulk density, γ_m_ of 881.3 and 673 kg per cubic meter (55 and 42 lbs per cubic foot), were assumed, and loads were tabulated at 1.524 m (5 feet) intervals of depth from the grain surface as shown in the Tables 1 and 2. For Janssen's load calculations, γ was taken as γ_0_. Currently, there is no technique to measure γ_m_, but an average bulk density over the entire storage bin can be calculated by using actual inventory mass in a particular grain bin.

**Table 1:** Comparison of vertical and horizontal stresses, *p*_z_ and *L*_z_ using Janssen's equations, and modified Janssen's equations proposed in this study for wheat

Depth, *z*	Janssen *p*_*z*_, kPa	Janssen *L*_*z*_, kPa	Modified *p*_*z*_, kPa	Modified *L*_*z*_, kPa	% Difference, *p*_*z*_	% Difference, *L*_*z*_
0	0	0	0	0	0	0
1.524	11.2	5.6	11.3	5.6	0.59	0.59
3.048	21	10.5	21.3	10.6	1.17	1.17
4.572	29.6	14.8	30.1	15.1	1.72	1.72
6.096	37.1	18.6	37.9	19	2.24	2.24
7.62	43.7	21.8	44.9	22.4	2.75	2.75
9.144	49.4	24.7	51	25.5	3.23	3.23
10.668	54.5	27.2	56.5	28.2	3.69	3.69
12.192	58.9	29.4	61.3	30.7	4.12	4.12
13.716	62.7	31.4	65.6	32.8	4.54	4.54
15.24	66.1	33.1	69.4	34.7	4.93	4.93
16.764	69.1	34.5	72.7	36.4	5.3	5.3
18.288	71.7	35.8	75.7	37.8	5.64	5.64
19.812	73.9	37	78.3	39.2	5.97	5.97
21.336	75.9	37.9	80.7	40.3	6.28	6.28
22.86	77.6	38.8	82.7	41.4	6.57	6.57
24.384	79.1	39.6	84.6	42.3	6.84	6.84
25.908	80.5	40.2	86.2	43.1	7.09	7.09
27.432	81.6	40.8	87.6	43.8	7.32	7.32
28.956	82.6	41.3	88.9	44.4	7.54	7.54
30.48	83.5	41.8	90	45	7.75	7.75
32.004	84.3	42.2	91	45.5	7.94	7.94
33.528	85	42.5	91.9	45.9	8.11	8.11
35.052	85.6	42.8	92.7	46.3	8.27	8.27
36.576	86.1	43.1	93.4	46.7	8.42	8.42
38.1	86.6	43.3	94	47	8.56	8.56

**Figure 2:**
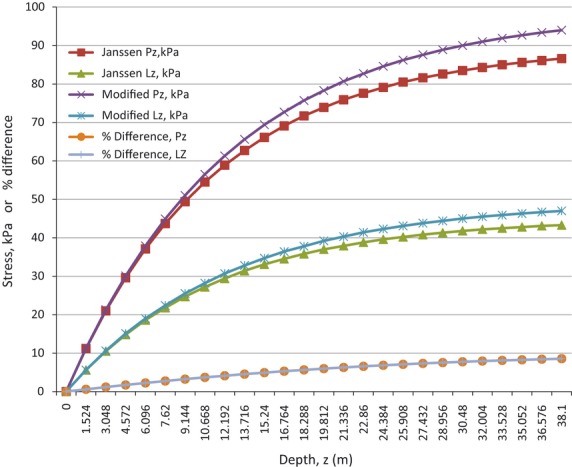
Comparison of vertical and horizontal modified stresses with corresponding Janssen stresses for wheat.

See Figure 2 along with this table. *p*_z_, vertical pressure of grain; *L*_z_, lateral pressure of grain.

**Table 2:** Comparison of vertical and horizontal stresses, *p*_z_ and *L*_z_ using Janssen's equations, and modified Janssen's equations proposed in this study for oats

Depth, *z* (m)	Janssen *p*_*z*_, kPa	Janssen *L*_*z*_, kPa	Modified *p*_*z*_, kPa	Modified *L*_*z*_, kPa	% Difference, *p*_*z*_	% Difference *L*_*z*_
0	0	0	0	0	0	0
1.524	7.2	3.6	7.3	3.6	1.57	1.57
3.048	13.4	6.7	13.9	6.9	3.1	3.1
4.572	18.9	9.5	19.8	9.9	4.58	4.58
6.096	23.7	11.9	25.2	12.6	6.03	6.03
7.62	28	14	30	15	7.43	7.43
9.144	31.6	15.8	34.4	17.2	8.78	8.78
10.668	34.9	17.4	38.4	19.2	10.08	10.08
12.192	37.7	18.8	42	21	11.34	11.34
13.716	40.2	20.1	45.2	22.6	12.54	12.54
15.24	42.3	21.2	48.1	24.1	13.7	13.7
16.764	44.2	22.1	50.7	25.4	14.8	14.8
18.288	45.9	22.9	53.1	26.6	15.85	15.85
19.812	47.3	23.7	55.3	27.6	16.86	16.86
21.336	48.6	24.3	57.2	28.6	17.81	17.81
22.86	49.7	24.8	59	29.5	18.72	18.72
24.384	50.7	25.3	60.6	30.3	19.58	19.58
25.908	51.5	25.8	62	31	20.39	20.39
27.432	52.2	26.1	63.3	31.6	21.16	21.16
28.956	52.9	26.4	64.5	32.2	21.88	21.88
30.48	53.5	26.7	65.5	32.8	22.56	22.56
32.004	54	27	66.5	33.2	23.2	23.2
33.528	54.4	27.2	67.3	33.7	23.79	23.79
35.052	54.8	27.4	68.1	34.1	24.35	24.35
36.576	55.1	27.6	68.8	34.4	24.9	24.9
38.1	55.4	27.7	69.5	34.7	25.37	25.37

**Figure 3:**
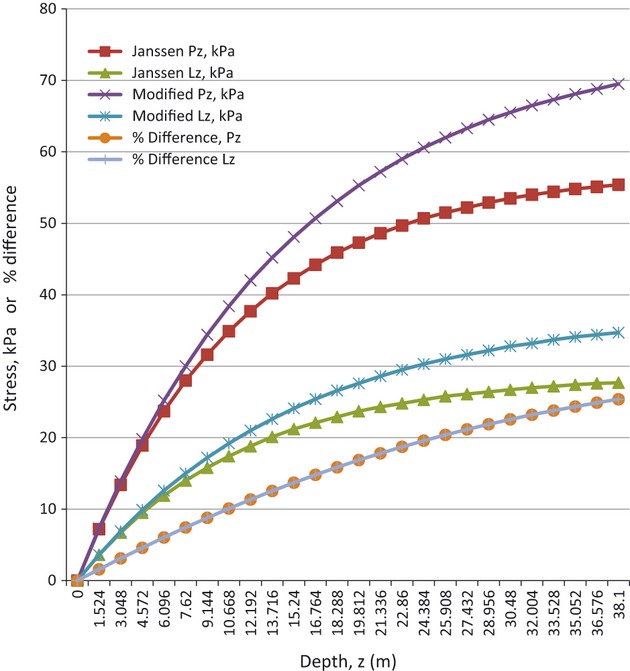
Comparison of vertical and horizontal modified stresses with corresponding Janssen stresses for oats.

See Figure 3 along with this table. *p*_*z*_, vertical pressure of grain; *L*_*z*_, lateral pressure of grain.

Using the noncompacted bulk densities in Janssen's equations to calculate vertical and horizontal stresses, Tables 1 (Fig. 2) and 2 (Fig. 3) clearly show that the modified Janssen's equations presented in this article consistently increased both load calculations for all depths of grains to account for the compaction of the grain beds due to the effects of changes in bulk densities. For the scenarios considered in these calculations and for wheat, the compaction effect was less compared with the oats and increased from 0% to 8.56%, but for oats, the increase was from 0% to 25.38%. For the same depth and same grain, the vertical and the horizontal pressures increased with the same rate, because the ratio of the lateral to vertical pressure is the constant, *k*.

## Conclusion

A mathematical model has been developed to describe a functional relationship between the specific weight of bulk solids and depth of packed bed. Principles of mathematics were used to prove the validity of the model developed. A law of compaction of granular materials is proposed. The law is stated as follows: “Each stored granular material has a minimum and a maximum limit to the compacted bulk density which are dependent on the storage system consisting of the properties of the material stored, storage container, and some operational parameters. The compacted bulk-density function increases exponentially with the depth of bed from the minimum to the maximum value with the rate of increase being equal to the rate of increase of static pressure imparted by the material at the same location.”

A modification of Janssen's equations has been proposed. The proposed change is based on the author's development of a mathematical equation relating bulk densities of grains stored in bins with the depth of bed. The example calculations presented in this article support a consistent increase of calculated pressures compared with Janssen's loadings.

The results of this study are supported by practical observations, that is, actual inventory of the mass of grain in a deep storage bin exceeds the calculated mass based on grain column dimensions and laboratory measurement of bulk density. The theory presented in this article could result in a method of predicting the actual mass of stored granular materials in bins more accurately than existing methods. This study could also guide the structural design engineers of bins to calculate vertical and horizontal stresses more accurately reflecting the effect of compaction of stored products in granular material storage bins.
